# EPA/DHA Concentrate by Urea Complexation Decreases Hyperinsulinemia and Increases Plin5 in the Liver of Mice Fed a High-Fat Diet

**DOI:** 10.3390/molecules25143289

**Published:** 2020-07-20

**Authors:** Alejandra Espinosa, Andrés Ross, Gretel Dovale-Rosabal, Francisco Pino-de la Fuente, Ernesto Uribe-Oporto, Camila Sacristán, Paulina Ruiz, Rodrigo Valenzuela, Nalda Romero, Santiago P. Aubourg, Alicia Rodríguez

**Affiliations:** 1 Department of Medical Technology, Faculty of Medicine, University of Chile, 8380000 Santiago, Chile; bespinosa@med.uchile.cl (A.E.); pinodelafuente.francisco@gmail.com (F.P.-d.l.F.); ernesto.uribe@live.com (E.U.-O.); c_sacristan@ug.uchile.cl (C.S.); paruiz@uchile.cl (P.R.); 2 Escuela de Medicina, Campus San Felipe, Universidad de Valparaíso, 2340000 Valparaíso, Chile; 3 Department of Food Science and Chemical Technology, Faculty of Chemical and Pharmaceutical Sciences, University of Chile, Santos Dumont 964, 8380494 Santiago, Chile; andres.ross.burrows@gmail.com (A.R.); gretel.dovale@ug.uchile.cl (G.D.-R.); nromero@uchile.cl (N.R.); 4 Nutrition Department, Faculty of Medicine, University of Chile, 8380000 Santiago, Chile; rvalenzuelab@med.uchile.cl; 5 Department of Food Technology, Marine Research Institute (CSIC); Eduardo Cabello, 6, 36208 Vigo, Spain; saubourg@iim.csic.es

**Keywords:** deodorized refined salmon oil, urea complexation, EPA + DHA concentration, metabolic alterations, insulin, obesity, perilipins

## Abstract

Dietary intake of eicosapentaenoic/docosahexaenoic acid (EPA/DHA) reduces insulin resistance and hepatic manifestations through the regulation of metabolism in the liver. Obese mice present insulin resistance and lipid accumulation in intracellular lipid droplets (LDs). LD-associated proteins perilipin (Plin) have an essential role in both adipogenesis and lipolysis; Plin5 regulates lipolysis and thus contributes to fat oxidation. The purpose of this study was to compare the effects of deodorized refined salmon oil (DSO) and its polyunsaturated fatty acids concentrate (CPUFA) containing EPA and DHA, obtained by complexing with urea, on obesity-induced metabolic alteration. CPUFA maximum content was determined using the Box–Behnken experimental design based on Surface Response Methodology. The optimized CPUFA was administered to high-fat diet (HFD)-fed mice (200 mg/kg/day of EPA + DHA) for 8 weeks. No significant differences (*p* > 0.05) in cholesterol, glycemia, LDs or transaminase content were found. Fasting insulin and hepatic Plin5 protein level increased in the group supplemented with the EPA + DHA optimized product (38.35 g/100 g total fatty acids) compared to obese mice without fish oil supplementation. The results suggest that processing salmon oil by urea concentration can generate an EPA+DHA dose useful to prevent the increase of fasting insulin and the decrease of Plin5 in the liver of insulin-resistant mice.

## 1. Introduction

Obesity is highly prevalent nowadays, being responsible for several comorbidities such as type-2 diabetes, hypertension, and cardiovascular diseases [1]. This pathophysiological condition is characterized by a lower level of systemic inflammation [2] and leads to an increase in basal proinflammatory mediators which affects the adequate insulin response in tissues such as skeletal muscle and adipose tissue, and produces negative effects on glucose homeostasis [3]. Inflammation of the adipose tissue produces the activation of Ser/Thr kinases such as Jun kinase (JNK) and tumor necrosis factor (TNF) by the immune system, which in turn inhibit the insulin intracellular signal transduction pathways [4,5]. The effect of insulin resistance in the liver is the impaired suppression of gluconeogenesis, which increases fasting glycemia. Hyperlipidemia can also be increased, because lipolysis cannot be suppressed by insulin in adipose tissue [6]. Insulin resistance is the previous step in type-2 diabetes development. Any dietary strategy to prevent or revert this condition can be considered as a good opportunity to gain time. Several studies support that diets enriched with eicosapentaenoic acid (EPA) and/or docosahexaenoic acid (DHA) improve hepatic insulin sensitivity in different models of mouse obesity and metabolic syndrome [7,8,9]. Dietary intake of omega-3 fatty acids produces a reduction in insulin resistance due to their effect on the expression and activity of enzymes that metabolize glucose, thus decreasing the expression of lipogenic enzymes and increasing the β-oxidation of fatty acids (FA) or the anti-inflammatory effect [10,11]. The anti-inflammatory actions of (C20:5 n-3, EPA) and (C22:6 n-3, DHA) are also crucial in the reported preventive mechanism. One of the most-used models of obesity is that of diet-induced obesity (DIO), which—within a few weeks—produces metabolic abnormalities such as insulin resistance and lipid accumulation intracellular lipid droplets in the liver [11,12]. This organelle is involved in the regulation of lipid metabolism, promoting β-oxidation of FA [13]. Perilipin 5 is a lipid droplet-associated protein (Plin) in this process, which has a relevant role; Plin2 is one of the proteins involved in the lipogenesis process and contributes to lipid droplet growth [13,14]. Currently, the main sources of EPA and DHA are commercial formulations based on oral ethyl esters [15], because the re-esterification process to obtain structured lipids implies high-cost methods. In this study, the effects of an EPA and DHA concentrate (CPUFA) obtained from fish viscera by urea complexation were studied. Considering that omega-3 FA (i.e., EPA and DHA) supplementation is a promising strategy to prevent several obesity-associated abnormalities, different biological markers altered by obesity in mice supplemented with the EPA-DHA concentrate were analyzed.

## 2. Results

### 2.1. Fatty Acid Composition and Quantification of Refined Salmon Oil

Table 1 shows the deodorized refined salmon oil (DSO) characterization by chemical analyses. Values obtained for peroxide value (PV), *p*-anisidine value (AV) and total oxidation (TOTOX) (0.29 ± 0.01, 3.70 ± 0.33 and 3.99 ± 0.34, respectively) revealed low lipid oxidation development, which agrees with previous research on different kinds of fish oils, including salmon oil [16,17,18,19]. Consequently, the present DSO oxidation values found are under the limits recommended for the human consumption (15 for AV value) [20].

DSO fatty acid composition and quantification revealed that the main fatty acids were oleic acid (C18:1 n-9), linoleic acid (C18:2 n-6), palmitic acid (C16:0); docosahexaenoic acid (C22:6 n-3, DHA); eicosapentaenoic acid (C20:5 n-3, EPA) with 38.90 ± 0.02, 14.68 ± 0.16, 11.40 ± 0.23, 3.57 ± 0.12 and 3.50 ± 0.06 g/100 g total fatty acids, respectively (Table 1). These results coincide with reported values [21].

### 2.2. Optimization of the Process for Obtaining CPUFA by Urea Complexation

DSO obtained under optimal conditions was subjected to urea complexation, applying a Box–Behnken design to optimize the EPA+DHA concentrate (CPUFA) yield. Table 2 presents the experimental conditions. The independent variables were urea/FA content ratio, crystallization temperature and crystallization time, as well as the values obtained for the response variables EPA content, DHA content and EPA + DHA content.

The analysis of variance (Table 3) showed that urea/FA content ratio (variable A), crystallization temperature (variable B) and crystallization time (variable C) in its linear form had a significant effect on EPA + DHA content (*p* < 0.05). AA, BB and CC in their quadratic form and interactions AB, AC, BC were also significant (*p* < 0.05). The model presented a standard error of 0.085 and a mean absolute error (MAE) of 2.267. The R^2^ statistic indicates that the model as fitted explains 90.895% of the variability in EPA DHA. The adjusted R^2^ statistic, which is more suitable for comparing models with different numbers of independent variables, was 74.507%.

### 2.3. Effect of Urea Concentration Process Variables on EPA, DHA and EPA + DHA of CPUFA: Analysis by RSM

**Figure 1:**
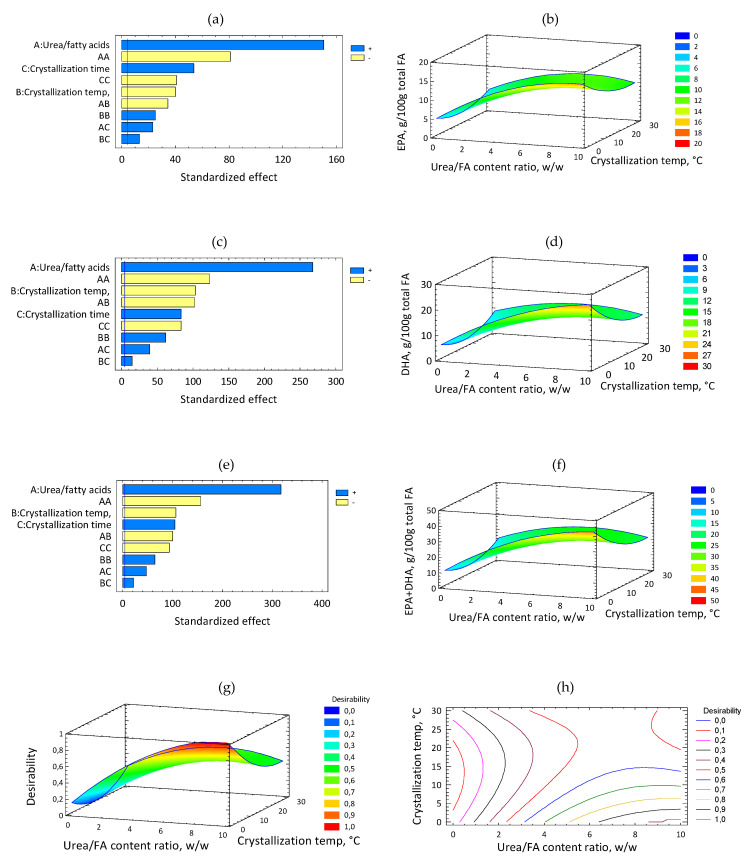
Effect of urea/fatty acid (FA) content ratio and crystallization temperature. Panels (**a**), (**c**), (**e**) (EPA, DHA, EPA+DHA standardized Pareto diagrams, respectively). Panels (**b**), (**d**), (**f**) (EPA, DHA, EPA+DHA response surface content (g/100 g total FA), respectively). (**g**) desirability function; (**h**) contours of estimated response surface.

The linear, quadratic and interaction terms in the second order polynomial were used to generate a three-dimensional response surface graph (Figure 1b,d,f,g).

The standardized Pareto diagram (Figure 1a) shows that all the linear variables, as well as their interactions, had significant effects (*p* < 0.05) on the EPA content. Figure 1b shows the response surface for EPA content as a function of the crystallization temperature and urea/FA content ratio. EPA content increased with the increase of urea/FA content ratio, while crystallization temperature led to a maximum content of EPA at low levels (*p* < 0.05). Figure 1c shows the standardized Pareto diagram for DHA content. All the independent variables and their interactions provided a significant effect on DHA content (*p* < 0.05). Figure 1d shows the response surface for DHA content as a function of the crystallization temperature and urea/FA content ratio. The DHA content increased with the urea/FA content ratio and reduced the crystallization temperature. This result agrees with the inverse relationship found between the urea/FA content ratio and crystallization temperature. The standardized Pareto diagram (Figure 1e) shows the effects of the independent variables on the response variable EPA + DHA content (the blue line indicates *p* < 0.05). Urea/FA content ratio was the most important factor in CPUFA, a higher urea/FA content ratio leading to an increase in EPA + DHA concentration. Figure 1f shows the response surface for EPA + DHA content as a function of the crystallization temperature and urea/FA content ratio. It can be observed that the urea: FA ratio variable led to a maximum content of EPA + DHA at high levels while the crystallization temperature had a negative effect (*p* < 0.05). Similar results were previously reported [21,22] and it can also be observed that final values of EPA + DHA in the present investigation are lower than those reported, due to the differences in the applied RSM methodology, since the current crystallization temperatures were higher, the number of experimental runs was lower and the initial raw material presented a lower initial concentration of EPA + DHA, since these were from different sources.

### 2.4. Optimization of the Process for Obtaining CPUFA and CDSO by Urea Complexation by RSM

Table 4 (A) shows the combination of factor levels which maximized the response variables on EPA + DHA concentrate (CPUFA) and EPA + DHA concentrate, similar to, deodorized refined salmon oil (CDSO) obtained by complexation urea with EPA+DHA similar content to DSO.

A ratio of 9.997 for urea/FA content ratio, a crystallization temperature of 4 °C and a crystallization time of 18.12 h was the combination that maximized the EPA + DHA production for CPUFA, thus leading to a maximum predicted value of 37.84 (g/100 g total FA) (Table 4 (A), Figure 1, Panels g and h). A value of 0.0447 for urea/FA content ratio, a crystallization temperature of 13.4 °C and a crystallization time of 17.28 h was the combination of the independent variables for CDSO similar to DSO, leading to a predicted value of 7.07 (g/100 g total FA) (Table 4 (A), Figure 1, Panels g and h).

Table 4 (B) shows the results of FA composition obtained for CPUFA and CDSO to validate the optimization process of urea complexation. In the FA composition validation, CPUFA obtained 38.35 (g/100 g total FA) of EPA + DHA content for the optimal value and 7.10 (g/100 g total FA) of EPA + DHA content for CDSO. Both validated and optimal values for CPUFA and CDSO were employed in the biological tests.

Table 4 (B) shows the PUFA composition of optimal concentrate with maximum EPA + DHA content of CPUFA and concentrate with EPA + DHA content, with a value similar to the concentrate of EPA+DHA with value of EPA + DHA similar to salmon oil (CDSO). The saturated and monounsaturated fatty acids were considerably reduced in the product optimized to maximum EPA + DHA, because these kinds of fatty acids form adducts with the crystallized urea and are retained in the aqueous fraction of the subsequent washes carried out in the process of complexation, in agreement with previously reported research [17,22,23,24,25,26,27]. In the complexation process, the DHA content of CPUFA reached higher amounts in the non-complex fraction, higher than that of EPA content, a situation previously reported in the literature, being explained on the basis of a lower tendency of DHA to form urea adducts given the difference in spatial conformational structure between these fatty acids [25,28,29,30].

### 2.5. Weights and Metabolic Parameters in High-Fat Diet (HFD)-Fed Mice

**Figure 2:**
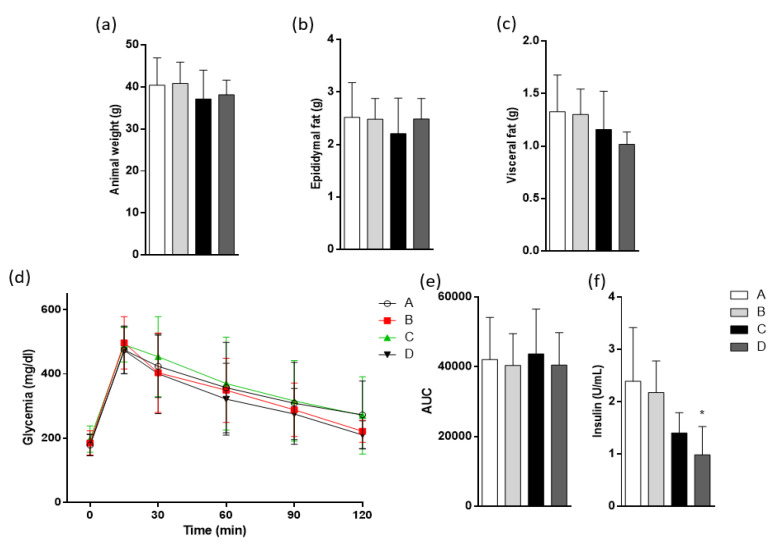
Effects of different products derived from fish oil on animal weight and the glucose homeostasis test. Animal groups were fed with high-fat diet (HFD) and divided into four groups. Oral oil administration: A, sunflower oil; B, DSO; C, CDSO concentrated by urea complexation and D, CPUFA Salmon oil concentrated by urea complexation. (**a**) Total animal weight at the final period of treatment, (**b**) epididymal fat weight, and (**c**) visceral fat weight. (**d**) Intraperitoneal glucose tolerance test (OGTT) assessed 8 weeks after treatments and the respective area under the curve (AUC) of OGTT. (**e**) Fasting serum insulin concentration. (**f**) Values are represented as mean ± SD, *n* = 8, * *p* < 0.05, by one-way analysis of variance (ANOVA) followed by Tukey post hoc test.

Total and fat weights were measured after 8 weeks of diet treatments. Group A was treated with sunflower oil as the control oil, without EPA/DHA, group B was treated with DSO containing 7.10 g (EPA + DHA)/100 g total FA) (Table 1), group C was treated with CDSO containing 7.10 g (EPA + DHA)/100 g total FA) (Table 4 (B)) and group D was treated with salmon oil concentrate (CPUFA) containing 38.35 g (EPA + DHA)/100 g total FA) (Table 4 (B)). The results (Figure 2a–c) show that total weight, visceral and epididymal fat of animals were similar in all four groups. The HFD-fed model used here is characterized by an impairment in glucose homeostasis Figure 2d,e, showing that none of the three treatments decreased the alteration in the oral glucose tolerance test observed in HFD-fed mice, but fasting serum insulin level was significantly lower in group D compared to group A (Figure 2f).

**Figure 3:**
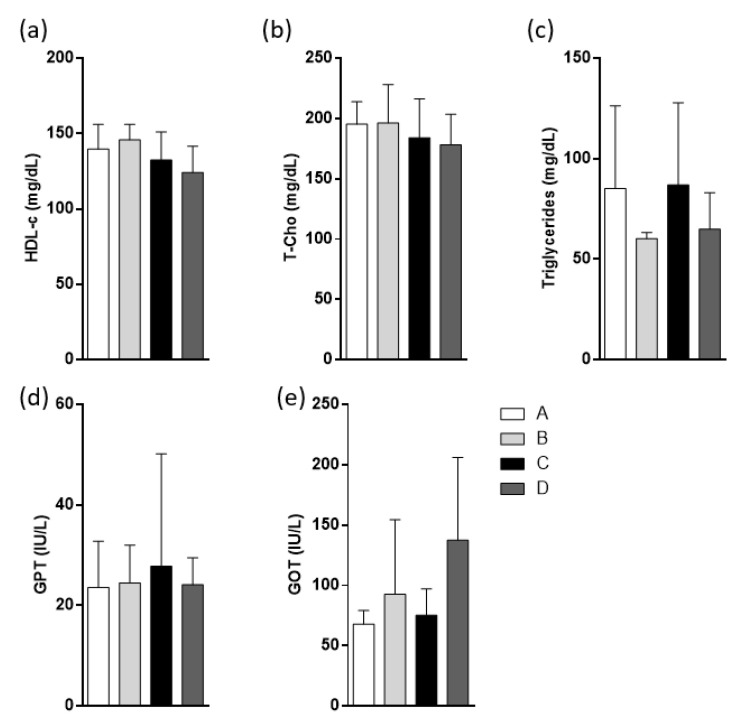
Effects of different products derived from fish oil on biochemical parameters. Animal groups were fed with HFD and divided into four groups. Oral oil administration was: A, sunflower oil; B, DSO; C, CDSO concentrated by urea complexation and D, CPUFA salmon oil concentrated by urea complexation. Fasting lipid profiles were measured; (**a**) serum HDL cholesterol concentration (HDL-c), (**b**) total cholesterol (T-Cho) levels, and (**c**) serum triglyceride concentration. Serum transaminases measured were: (**d**) serum glutamic oxaloacetic transaminase (GOT) and (**e**) glutamic pyruvic transaminase (GPT). All biochemical measurements were made after 4 h of fasting. Values are represented as mean ± SD, *n* = 8, * *p* < 0.05, by one-way analysis of variance (ANOVA) followed by Tukey post hoc test.

The biochemical analysis was also performed in the serum of mice, but no significant differences were found in fasting lipid profiles (Figure 3a–c) among groups. Transaminase levels were measured to evaluate possible hepatic damage. No significant differences in transaminases were found among groups (Figure 3d,e) or in transaminase measurements.

### 2.6. Fish Oil Products do Not Affect the Lipid Droplets (LD) Oxidation Induced by HFD

**Figure 4:**
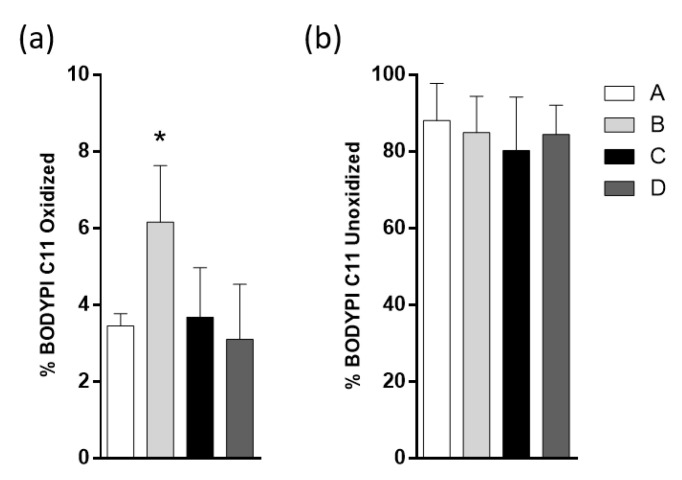
Effect of different products derived from fish oil on the intracellular lipid content present in freshly isolated hepatocytes from high-fat diet (HFD)-fed mice. Animal groups were fed with HFD and divided into four groups. Oral oil administration was: A, sunflower oil; B, DSO; C, CDSO concentrated by urea complexation and D, CPUFA salmon oil concentrated by urea complexation. The hepatocytes isolated from each group were selected by Forward scatter/ Side scatter (FSC/SSC) gating analysis. Subsequently, hepatocyte BODIPY C11 (+) (red cells, PE filter) were gated, then positive green cells were selected (BODIPY C11 oxidized (+) cells) (FITC filter) (**a**) Quantification of the percentage of hepatocytes C11+. (**b**). Quantification of the percentage of C11-oxidized hepatocytes in HFD-fed mice treated with different products derived from fish oil. Data are presented as mean ± SEM; *n* = 3 mice for each condition, * *p* < 0.05, by Mann–Whitney test.

One of the main alterations in the current obesity model is the increase in liver lipid content, which increases both the size and number of LDs [8,12]. HFD-fed mice also presented a higher percentage of oxidized lipids within the LDs with respect to non-obese mice. The fluorescence intensity of the BODIPY C11 probe in isolated hepatocytes was measured using flow cytometry. Levels of total labeled hepatocytes were similar among the four groups, indicating that lipids in the hepatocytes were not modified (Figure 4a). BODIPY C11 was found to shift from red to green upon oxidation, the green fluorescent label was measured and showed that the DSO group had the highest percentage of oxidized hepatocytes (Figure 4b). There is an observable tendency to decrease oxidized hepatocyte levels in group D compared to B.

### 2.7. Plin5 Protein Level Increase with Dietary Fish Oil Products Intake in HFD-Fed Mice

**Figure 5:**
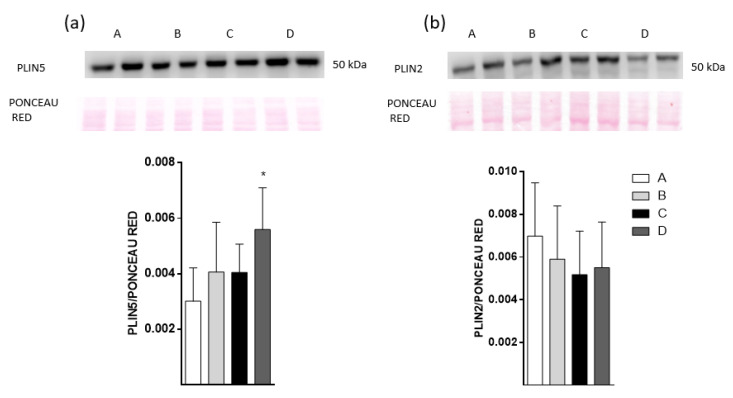
Perilipin levels in freshly isolated hepatocytes from high-fat diet (HFD)-fed mice. Animal groups were fed with HFD and divided into four groups. Oral oil administration: A, sunflower oil; B, DSO; C, CDSO concentrated by urea complexation and D, CPUFA Salmon oil concentrated by urea complexation. Lysates from isolated hepatocytes were used to determine Plin5 (**a**) and Plin2 (**b**) protein levels by Western blot. The top image shows the original gel plus the respective Ponceau red stain, and the bottom image shows the densitometric analysis. Data are presented as mean ± SEM; * *p* < 0.05, by one-way analysis of variance (ANOVA) followed by Tukey post hoc test.

To evaluate some of the LD dynamics in the liver of HFD-fed mice, the Plin2 and Plin5 protein levels were measured based on being representative proteins associated with lipogenesis and lipolysis processes, respectively (Figure 5). The results show that group D had the highest level of Plin5 among the four groups. The Plin2 protein level was not different among groups.

## 3. Discussion

This study assessed the effect of different fish oil preparations on some of the obesity-metabolic alterations induced by HFD. Characterization, fatty acid composition and quantification of DSO processing were similar to those reported by others, showing a low lipid oxidation stage as with other fish oils [16,18]. The present results indicate levels of EPA and DHA of 3.50 ± 0.06 g/100 g total FA and 3.57 ± 0.12 g/100 g total FA, respectively, lower values than those previously reported, illustrating the substantial substitution of marine ingredients with vegetable ingredients in the feed of farmed salmon in the last decades [31,32]. The level of linoleic acid (C18:2 n-6) is particularly high (14.68 ± 0.16 g/100 g total FA) compared to the usual values reported for wild salmon, which accounts for about 1 g/100 g total FA and is recognized as a good indicator that vegetable oil has been fed to the fish [31]. 

Our results are also in line with previous reports, where the efficiency of complexation is higher at low crystallization temperatures and high ratios of urea:FA [18,21,22,33], showing that the tendency of FA to combine with urea crystals to form adducts decreases as the unsaturation levels and chain lengths increase, which is evidence of an inverse relationship between the urea:FA content ratio and the crystallization temperature.

The effect of these products on different metabolic parameters classically affected in obesity was also analyzed. The HFD model induced an obese phenotype as has been reported before [34,35]. Physiological measurements show that the different fish oil products have a discrete impact on mouse physiology. Our low performance probably was due to the low concentration obtained compared to other studies such as Valenzuela et al. [11], where doses of 108 mg/kg EPA and 92 mg/kg DHA were administrated daily to HFD-fed mice for three months. They had a significant impact on animal weight and homeostasis glucose parameters. In our study, a significant impact on the fasting insulin level was obtained using the optimized salmon oil concentrated by urea complexation.

Fasting insulin levels reflect the liver management of glucose in the fasting period; a prediabetes condition is characterized by an increase of fasting insulin with high insulin levels, impaired fasting glucose and impaired glucose tolerance [36,37]. Our results support that the administration of EPA and DHA concentrate from fish oil using RSM is capable of reducing the obesity-associated hyperinsulinemia. Although the three kinds of EPA/DHA supplementation did not improve the lipid profile, neither lipidic hepatocyte content nor oxidized LD content are classically altered in HFD-fed mice [11,34]. The statistical result of Figure 4b shows that the highest oxidized group was B, while groups C and D showed a lower level of oxidized lipids in hepatocytes. This result agrees with the reported role of EPA/DHA supplementation in the prevention of oxidative stress in non-alcoholic liver disease by NRF2 up-regulated antioxidant enzymes [38,39].

In this study, we demonstrate that the administration of 38.35 g/100g total fatty acids did not induce liver injury, as we showed Figure 3d,e. Some reports have shown that EPA/DHA supplementation induces the hepatic damage when the metabolic alterations have been onset. However, in these studies, higher fish oil doses than we used or other mice strains were used, which makes those studies not comparable with our results [13,40,41].

Plin5 increased in hepatocytes from HFD-fed mice treated with the EPA/DHA concentrate by urea complexation process. The fact that Plin5 is involved in intrahepatic lipidic metabolism, allowing the interaction between LDs-mitochondria to enable lipid interchange, is a remarkable finding of our study. Liver-specific Plin5-deficient mice develop less lipidic oxidation and higher lipid storage than control mice [42]. These mice have both poor glycemic control and insulin resistance, probably due to the unregulated intracellular lipid content through live [42]. Overexpression of PLIN5 was associated with reduced serum insulin levels [43]. This last work is consistent with our results, where we showed that mice treated with salmon oil concentrate by urea complexation had less insulin level than HFD-fed mice.

It could be attractive to continue exploring how the machinery involved in lipolysis is affected by the current doses of EPA/DHA; still, more studies are necessary from a biological point of view.

## 4. Materials and Methods

### 4.1. Materials

Sunflower oil (*Helianthus annuus*), first cold pressed (SO), Natura (Aceitera General la Dehesa), containing saturated fat 9.4%, monounsaturated fat 32.2%, polyunsaturated fat 49.6%, Vitamin E and no EPA/DHA was purchased from a supermarket (Líder, Santiago, Chile). Deodorized refined salmon oil (DSO) from the Atlantic salmon (*Salmo salar*), rainbow trout (*Oncorhynchus mykiss*) and Pacific salmon (*Oncorhynchus kisutch*) and antioxidant-free was provided by Natural Oils Chile S. A. (Santiago, Chile). The internal standard for gas chromatography (GC) analysis, tricosanoic acid methyl ester (C23:0) was obtained from Matreya LLC—USA. Hexane and toluene used for GC analysis were HPLC grade and obtained from Merck S.A. (Santiago, Chile).

### 4.2. DSO Characterization

Initial DSO was characterized by different physical and chemical analyses. Standard AOCS official method procedure (AOCS, 1993) was employed for the following assessments: free fatty acid (FFA) content (method Ca 5a–40:1), peroxide value (PV; method Cd 8b–90:1–2), *p*-anisidine value (AV; method Cd 18–19:1–2) and total oxidation (TOTOX) value.

### 4.3. Fatty Acid Composition and EPA/DHA Quantification

The DSO fatty acid profile and EPA/DHA quantification were assessed in a PerkinElmer Clarus 680 GC Chromatograph (PerkinElmer, Boston, MASS, USA) with flame ionization detector. A Perkin Elmer Elite Wax Capillary Column (Polyethylene Glycol Phase, PerkinElmer, Boston, MASS, USA), 30 m × 0.25 mm × 0.25 um was used. Analytical standards: FAME Mix 37, Supelco-Bellefonte USA; n-3 PUFA Menhaden oil and PUFA -1, Supelco-Bellefonte-USA; methyl tricosanoate Matreya LLC-USA, (C23: 0 methyl ester 100 mg), eicosapentaenoic acid ethyl ester CRS Matreya LLC-USA, (C20:5 n-3 ethyl ester 1 g) docosahexaenoic acid ethyl ester CRS Matreya LLC-USA, (C22:6 n-3 methyl ester 150 mg). The identification of fatty acids was performed by comparing the retention times of the analytical standards (Phr. Eur 2.4.29). The integration of the chromatographic peaks was carried out from baseline to baseline.

### 4.4. EPA and DHA Concentrate from DSO by Urea Complexation Process (CPUFA)

The preparation of EPA and DHA free fatty acid (FA) concentrates from DSO was carried out by the saponification and urea inclusion method [18,21]. The basic hydrolysis of DSO was performed according to Wanasundara and Shahidi [30] and Guil-Guerrero and Belarbi [23], with some modifications [17].

### 4.5. CPUFA Concentrate

For each experimental run, 40.0 g of FA was used with the urea previously dissolved in 95% ethanol (1:3.7 ratio based on the weight of the urea). The urea/FA content ratio was established according to each essay of the experimental design (Table 2). The FA/ethanolic urea solution mixture was heated to 60 °C in a low reflux system with constant stirring until completely dissolved. The solution was cooled to room temperature and the subsequent crystallization stage. At the end of this stage, the urea-FA adducts were also separated from the uncomplexed fraction by vacuum filtration with a Whatman No. 1 filter. The uncomplexed fraction was diluted with 400 mL distilled water and acidified to pH 4.5 with 6N HCl. The uncomplexed fraction was washed twice with 200 mL hexane each time in a decantation funnel and the hexane phases obtained were filtered in the presence of anhydrous Na_2_SO_4_. The solvent was evaporated in vacuum at 47 °C on a rotary evaporator. The EPA + DHA concentrate product was stored at −80 °C without adding antioxidant.

### 4.6. Optimization of CPUFA by Response Surface Methodology (RSM)

The Box–Behnken experimental design based on RSM was used where the effect of the three independent variables were studied; urea concentration:free fatty acids ratio (Urea/FA content ratio, 0–10 *w*/*w*) (variable A), crystallization temperature (4–25 °C) (variable B), crystallization time (0–24 h), (variable C) on the variables response concentration of EPA (g/100 g total FA), DHA (g/100 g total FA) and EPA + DHA (g/100 g total FA) (CPUFA) (Table 2). The design included 15 total experimental runs, three of them corresponding to central points that allowed estimating the experimental error. The experiments were performed in a randomized way to minimize the effect of variability on the observed responses.

Optimization of the EPA and DHA concentrate was performed using RSM [43,44]. To quantify the EPA and DHA content (g/100 g total FA) of each EPA + DHA concentrate from the 15 experimental runs, a (Gas chromatography) GC was performed according to the protocol of the Phr. Eur 2.4.29 [45]. The data allowed us to build predictive quadratic polynomial models in terms of their regression coefficients for the independent variables and establishing the combination of the variables that obtained a theoretical or predicted optimum of optimal EPA+DHA concentrate with a maximum content of EPA and/or DHA. A mathematical model was obtained from the RSM, with which the effect of the independent variables set could be predicted:

(1) $$Yi=\beta 0+\overset{4}{\underset{i=1}{\sum }}\beta iXi+\overset{4}{\underset{i=1}{\sum }}\beta iiXi^{2}+\overset{4}{\underset{i=1}{\sum }}\beta ijX_{i}X_{j}+\varepsilon$$

β0, βi, βii and βij represent the intercept, linear, quadratic and interaction regression coefficients, respectively, and Xi and Xj the independent variables. The regression coefficients were obtained by multiple regression analysis, using a significance level of *p* < 0.05. An ANOVA of the regression parameters and the fitted model was performed with a significance level of *p* < 0.05. The Statgraphics Centurion XVI-2011 statistical program (Stat Point Technologies, Inc., Rockville, MD, USA) was used. Finally, the EPA/DHA concentrate was experimentally validated by applying the conditions proposed in the theoretical optimum. The optimal EPA + DHA concentrate obtained was stored under a nitrogen atmosphere without antioxidant addition until use.

### 4.7. Animals

Male mice (C57BL/6, 6 weeks of age, 20.0 ± 2.0 g) were obtained from Instituto de Salud Pública, Chile. All mice were housed in a temperature-controlled room with a 12 h:12 h light/dark cycle and were given ad libitum access to their specific diet and water.

### 4.8. Biological Samples

Animals were randomly divided into four groups. Male C57BL/6 adult mice were fed with HFD (60% fat, 20% protein and 20% carbohydrates) and orally supplemented with 150 µL oil as follows: (a) sunflower oil, without EPA/DHA (control condition); (b) DSO, 7.10 g (EPA + DHA)/100 g total FA (Table 1); DSO concentrate by urea complexation, CDSO, 7.10 g (EPA + DHA)/100 g total FA (Table 4 (B)) and (d) salmon oil concentrate by urea complexation, CPUFA 38.35 g (EPA + DHA)/100 g total FA. Doses were administered daily for 8 weeks. Mice had free access to diets and water throughout the study. Animals were maintained in accordance with the NIH Guide for the Care and Use of Laboratory Animals and protocols were approved by the bioethical committee of the Faculty of Medicine, University of Chile (bioethical protocol number CBA1001, FMUCH, April 2018 approved). At the end of the experimental period, mice were anesthetized (isoflurane 5%) after four hours of fasting. Serum was collected after centrifugation of the blood (2000× *g* at 4 °C for 15 min), and biochemical determinations were made immediately.

### 4.9. Measurements of Serum Parameters

Levels of total cholesterol, triacylglycerols, alanine aminotransferase (ALT), aspartate aminotransferase (AST) and alkaline phosphatase (ALP) were measured by dry chemistry technology (SPOTCHEM™ EZ, Minneapolis, USA). Serum insulin concentrations were determined by a commercially available immunoassay specific for mice (Mercodia, Uppsala, Sweden).

### 4.10. Glucose Tolerance Test

An intraperitoneal glucose tolerance test (IGTT) was performed after four h of fasting. Basal fasting was taken as 0 min. Blood samples were obtained 15, 30, 60 and 120 min after intraperitoneal injection of glucose from the tail vein.

### 4.11. Hepatocytes Isolation and Flow Cytometry

For hepatocyte isolation, 4–5 mm samples were mechanically disaggregated using a Tissue Tearor Homogenizer (Cole-Parmer, Vernon Hills, IL, USA) in PBS 0.1X to remove red blood cells. Hepatocytes were purified by density gradient centrifugation in Ficoll (Sigma, St. Louis, MO, USA); after centrifugation at 2660× *g* for 90 s the hepatocyte pellet was washed 3 times with PBS. This preparation was used to perform immunofluorescence and lipoperoxidation labeling analysis with 4,4-difluoro-5-(4-phenyl-1,3-butadienyl)-4-bora-3a,4a-diaza-s-indacene-3-undecanoic acid (BODIPY^®^ 581/591 C11) (Invitrogen, Carlsbad, CA, USA) as reported previously [34].

Hepatocytes were incubated with BODIPY 581/591 for 60 min at room temperature. The fluorescence in the cells was analyzed using flow cytometry. Then they were rinsed twice with PBS and measured on a FACSCanto II flow cytometer (BD Biosciences, San Jose, CA, USA)). Data analysis was performed using the FlowJo software (Tree Star, Inc., Ashland, OR, USA).

### 4.12. Western Blot Analysis of Perilipins

Isolated hepatocytes were homogenized in lysis buffer and protease inhibitors (1 mM phenylmethylsulfonyl fluoride, 1 µg mL^−1^ aprotinin, 1 µg mL^−1^ leupeptin and 1mM orthovanadate). Proteins were separated on 10% using SDS-PAGE and then transferred to PVDF membranes. Ponceau 4R was used as internal control in all determinations. Incubation with anti-Plin2 (#PA5-79830, Invitrogen, 1:1000) and anti-Plin5 (#PA1-46215, Invitrogen, 1:1000) was performed at 4 °C overnight and secondary antibody treatment for 1 h at room temperature.

### 4.13. Statistics

A statistical analytical system was used for multiple regression analysis, analysis of variance (ANOVA), canonical analysis and analysis of ridge maximum of data in the response surface regression (RSREG) procedure. Estimated response surfaces and contours of estimated response surface were developed using the fitted quadratic polynomial equations obtained from RSREG analysis and holding the process variables with the least effect on the response at a constant value and changing the levels of the other two variables. Analyses were performed in triplicate. The 95% confidence intervals of each quality parameter were calculated, considering the number of replicates and the standard deviation of each sample. The lack-of-fit test was performed by comparing the variability of the current model residuals with the variability between observations at replicate settings of the factors. Statgraphics ^®^Centuriun XVI-2011 software (StatPoint Technologies, Inc., Rockville, USA) was used. Analysis of variance (ANOVA) was used in biological test (Tukey post hoc, Figure 2 and Figure 3, Mann–Whitney, Figure 4 and Figure 5).

## 5. Conclusions

The optimal EPA + DHA concentrate obtained from salmon oil can be developed using Response Surface Methodology (RSM) with biological activity. The main effects of this optimized oil were the prevention of hyperinsulinemia in an obese mouse model. Additionally, Plin5 increased in HFD-fed mice, showing a potential product for the prevention of LD dysregulation in the liver of obese mice.

## Figures and Tables

**Table 1:** Characterization, fatty acid (FA) composition and quantification of deodorized refined salmon oil (DSO).

Systematic Name	Abbreviated Name	DSO (g/100 Total FA)
Myristic acid	C14:0	2.17 ± 0.07
Palmitic acid	C16:0	11.40 ± 0.23
Palmitoleic acid	C16:1 n-7	3.29 ± 0.06
Hexadecadienoic acid	C16:2 n-4	0.29 ± 0.01
Hexadecatrienoic acid	C16:3 n-4	0.26 ± 0.01
Hexadecatetraenoic acid	C16:4 n-1	0.42 ± 0.01
Heptadecanoic	C17:0	0.03 ± 0.01
10c-heptadecenoic	C17:1	0.08 ± 0.05
Stearic acid	C18:0	3.19 ± 0.05
Oleic acid	C18:1 n-9	38.90 ± 0.02
cis-vaccenic acid	C18:1 n-7	3.23 ± 0.03
Linoleic acid	C18:2 n-6	14.68 ± 0.16
Linolenic acid (alpha)	C18:3 n-3	4.11 ± 0.02
Linolenic acid (Gamma)	C18:3 n-6	0.18 ± 0.01
Stearidonic acid	C18:4 n-3	0.72 ± 0.01
Arachidic acid	C20:0	0.29 ± 0.01
Gadoleic acid	C20:1 n-11	0.19 ± 0.01
Eicosenoic acid	C20:1 n-9	1.99 ± 0.02
Paullinic acid	C20:1 n-7	0.11 ± 0.01
Eicosadienoic acid	C20:2	0.09 ± 0.01
Eicosatrienoic acid	C20:3 n-3	0.27 ± 0.01
Dihomo-gamma-linolenic acid	C20:3 n-6	0.86 ± 0.02
Eicosatetraenoic acid	C20:4 n-3	0.59 ± 0.02
Arachidonic acid	C20:4 n-6	0.29 ± 0.01
Eicosapentaenoic Acid (EPA)	C20:5 n-3	3.50 ± 0.06
Heneicosapentaenoic acid	C21:5 n-3	0.23 ± 0.02
Erucic acid	C22:1 n-9	0.34 ± 0.02
Docosapentaenoic acid	C22:5 n-3	1.53 ± 0.03
Docosapentaenoic acid	C22:5 n-6	0.15 ± 0.04
Docosahexaenoic Acid (DHA)	C22:6 n-3	3.57 ± 0.12
Nervonic acid	C24:1 n-9	0.20± 0.01
Total Fatty acid Omega-3		14.5 ± 0.25
Total identified		97.94 ± 0.02
Total unidentified		2.06 ± 0.02
Peroxide value (PV), (meq active oxygen kg^−1^ oil)		0.29 ± 0.01
*p*-Anisidine Value (AV) Totox Value		3.70 ± 0.33 3.99 ± 0.34

**Table 2:** Box–Behnken design variables to optimize the eicosapentaenoic acid + docosahexaenoic acid (EPA + DHA) concentration process (CPUFA).

Independent and Response Variables of CPUFA						
Run	Urea/FA Content Ratio	Crystallization Temperature (°C)	Crystallization Time (h)	EPA (g/100 g Total FA)	DHA (g/100 g Total FA)	EPA + DHA (g/100 g Total FA)
1	0	4.0	12	3.12	3.41	6.53
2	10	4.0	12	16.38	25.08	41.46
3	0	25.0	12	3.14	3.40	6.54
4	10	25.0	12	11.24	13.12	24.36
5	0	14.5	0	3.29	3.57	6.86
6	10	14.5	0	6.87	7.76	14.63
7	0	14.5	24	3.16	3.61	6.77
8	10	14.5	24	10.23	12.4	22.63
9	5	4.0	0	9.32	11.94	21.26
10	5	25.0	0	6.67	8.46	15.13
11	5	4.0	24	12.42	15.63	28.05
12	5	25.0	24	11.74	13.89	25.63
13	5	14.5	12	10.73	13.09	23.82
14	5	14.5	12	10.65	13.20	23.85
15	5	14.5	12	10.58	13.11	23.69

**Table 3:** Analysis of variance for the variable response EPA + DHA concentrate (CPUFA)

Independent Variables	Sum of Squares	Medium Square	F	*p*-Value
A: Urea/FA content ratio	729.238	729.238	100,816.32	0.0000
B: Crystallization temp.	82.1762	82.1762	11,360.76	0.0001
C: Crystallization time	79.38	79.38	10,974.19	0.0001
AA	177.301	177.301	24,511.71	0.0000
AB	73.188	73.188	10,118.16	0.0001
AC	16.362	16.362	2262.03	0.0004
BB	30.3161	30.3161	4191.17	0.0002
BC	3.44102	3.44102	475.72	0.0021
CC	63.1192	63.1192	8726.15	0.0001
Lack of fit	126.111	42.0371	5811.58	0.0002
Pure error	0.0144667	0.00723333		
Total (corr.)	1385.28			

**Table 4:** Optimal values for CPUFA, deodorized salmon oil concentration (CDSO) and validation.

**A. Optimal Values for Maximum CPUFA and Optimal Values for CDSO**		
	**CPUFA: Maximize EPA + DHA** **Optimum Value = 38.35** **(g/100 g total FA)**	**CDSO: EPA + DHA Similar Content to DSO** **Optimum value = 7.10** **(g/100 g total FA)**
**Independent Variables**	**Process Optimum Variables**	**Process Optimum Variables**
Urea/FA content ratio	9.997	0.047
Crystallization temp, °C	4.000	13.400
Crystallization time, h	18.120	17.380
**B. Validation of CPUFA and CDSO by Fatty Acid Composition**		
FA	CPUFA (g/100 g total FA)	CDSO (g/100 g total FA)
C14:0	0.03	2.17
C16:0	0.25	11.25
C18:0	0.14	4.11
C18:1	1.70	40.76
C18:2	19.08	14.7
C18:3	8.58	4.43
EPA	15.80	3.34
DHA	22.55	3.76
EPA + DHA	38.35	7.10
